# A Review of Accelerometry-Based Wearable Motion Detectors for Physical Activity Monitoring

**DOI:** 10.3390/s100807772

**Published:** 2010-08-20

**Authors:** Che-Chang Yang, Yeh-Liang Hsu

**Affiliations:** Department of Mechanical Engineering, Yuan Ze University / 135, Yuan-Tung Rd., Chung-Li, 32003, Taiwan; E-Mail: s958702@mail.yzu.edu.tw

**Keywords:** accelerometry, accelerometer, physical activity, human motion, energy expenditure, gait, fall detection

## Abstract

Characteristics of physical activity are indicative of one’s mobility level, latent chronic diseases and aging process. Accelerometers have been widely accepted as useful and practical sensors for wearable devices to measure and assess physical activity. This paper reviews the development of wearable accelerometry-based motion detectors. The principle of accelerometry measurement, sensor properties and sensor placements are first introduced. Various research using accelerometry-based wearable motion detectors for physical activity monitoring and assessment, including posture and movement classification, estimation of energy expenditure, fall detection and balance control evaluation, are also reviewed. Finally this paper reviews and compares existing commercial products to provide a comprehensive outlook of current development status and possible emerging technologies.

## 1. Introduction

Physical activity (PA) is regarded as any bodily movement produced by skeletal muscles which results in an energy expenditure [1]. PA has been studied in epidemiological research for investigating human movements and the relationship to health status, especially in the area of cardiovascular diseases, diabetes mellitus and obesity. A declining PA level represents a major factor in multiple illnesses and symptoms related to functional impairment [2]. The organization Healthy People 2020 [http://www.healthypeople.gov/HP2020/] led by the U.S. government has recognized PA as one of the leading health indicators (LHI), which are a measurement of health of a nation’s population.

Various methods of subjective and objective PA assessment tools have been developed. Subjective methods, such as diaries, questionnaires and surveys, are inexpensive tools. However, these methods often depend on individual observation and subjective interpretation, which make the assessment results inconsistent [3]. Some standard tests for PA assessment also require subjective judgments. For example, the timed up-and-go test (TUG-T) is a simple test for evaluating one’s ability to perform a sequence of basic activities, and the result of the TUG-T can be a predictor for risk of falling [4]. Distinguishing postural transitions in the TUG-T, however, depends on subjective judgment that counts the time taken for each posture transition. The Berg Balance Scale (BBS), a valid measure to evaluate balance control of the elderly individuals, also requires subjective observation and determination for scoring some test items [5].

On the other hand, objective techniques use wearable, or body-fixed motion sensors, which range from switches, pedometers, actometers, goniometers, accelerometers and gyroscopes, for PA assessment. Mechanical pedometers, or so-called “step counters”, are the simplest wearable sensors to measure human motion. The pedometer uses a spring-loaded mass or some other switch mechanism to detect the obvious impacts produced by steps during locomotion. The number of steps during motion can be registered to estimate the distance walked and the energy expenditure. Though pedometers are cheap and simple, the major drawbacks are that pedometers cannot reflect intensity of movement and therefore result in inaccurate energy expenditure estimations [6]. PA can also be objectively measured by means of magnetic systems, optical systems, or video recording. Magnetic and optical systems for PA monitoring are costly and require complex instrumentation and environment setting. Privacy concerns are a major drawback in monitoring systems based on video recording. These systems may not be practical for monitoring subjects in free-living environments.

Accelerometers are sensors which measure the accelerations of objects in motion along reference axes. Measuring PA using accelerometry is preferred because acceleration is proportional to external force and hence can reflect intensity and frequency of human movement. Accelerometry data can be used to derive velocity and displacement information by integrating accelerometry data with respect to time [7]. Some accelerometers can respond to gravity to provide tilt sensing with respect to reference planes when accelerometers rotate with objects. The resulting inclination data can be used to classify body postures (orientations). With these characteristics, accelerometry is capable of providing sufficient information for measuring PA and a range of human activities. Accelerometers have been widely accepted as useful and practical sensors for wearable devices to measure and assess PA in either clinical/laboratory settings or free-living environments [8].

Accelerometers were first investigated in the 1950s to measure gait velocity and acceleration [9]. Accelerometry measurement of human motion was studied in more detail during the 1970s due to technological advances [10]. It was also shown that accelerometers had advantages over other techniques in quantitatively measuring human movement. Micro-electromechanical system (MEMS) technology has reduced the cost of accelerometers in smaller form factors. In the meantime, sensor performance has been enhanced while the power consumption is greatly reduced. The first batch-fabricated MEMS accelerometers were reported in 1979 [11]. Since then various research and commercial applications have used MEMS accelerometers in wearable systems for PA monitoring.

This paper provides a comprehensive review on the working principles, capabilities, and various applications of accelerometry-based wearable motion detectors for PA monitoring and assessment. The authors searched for published literature after year 2000 using a range of related keywords such as “accelerometry”, “accelerometer”, “wearable”, “physical activity”, “human motion”, “human movement”, “activity classification”, “energy expenditure”, “fall detection”, “balance stability” and “gait”. Selected literatures before year 2000 are also included. This paper first discusses the principles and fundamentals of accelerometry, along with different sensor placements. Various research using accelerometry-based wearable motion detectors for PA monitoring and assessment, including posture and movement classification, estimation of energy expenditure, fall detection and balance control evaluation, are then reviewed. Finally this paper reviews and compares existing commercial products to provide a comprehensive outlook of current development status and possible emerging technologies.

## 2. Design Fundamentals for Accelerometry-Based Wearable Motion Detectors

### 2.1. Accelerometry: Principles and Sensors

Inertial sensors are basically force sensors to sense linear acceleration along one or several directions, or angular motion about one or several axes. The former is referred to as an accelerometer, and the later a gyroscope. The common operation principle of accelerometers is based on a mechanical sensing element which consists of a proof mass (or seismic mass) attached to a mechanical suspension system with respect to a reference frame. Inertial force due to acceleration or gravity will cause the proof mass to deflect according to Newton’s Second Law. The acceleration can be measured electrically with the physical changes in displacement of the proof mass with respect to the reference frame. Piezoresistive, piezoelectric and differential capacitive accelerometers are the most common types [12,13].

#### 2.1.1. Piezoresistive accelerometers

The sensing element consists of a cantilever beam and its proof mass is formed by bulk-micromachining. The motion of the proof mass due to acceleration can be detected by piezoresistors in the cantilever beam and proof mass. The piezoresistors are arranged as a Wheatstone bridge to produce a voltage proportional to the applied acceleration. Piezoresistive accelerometers are simple and low-cost. The piezoresistive accelerometers are DC-responsive that can measure constant acceleration such as gravity. The major drawbacks of piezoresistive sensing are the temperature-sensitive drift and the lower level of the output signals.

#### 2.1.2. Piezoelectric accelerometers

In a piezoelectric accelerometer, the sensing element bends due to applied acceleration which causes a displacement of the seismic mass, and results in an output voltage proportional to the applied acceleration. Piezoelectric accelerometers do not respond to the constant component of accelerations.

#### 2.1.3. Differential capacitive accelerometers

The displacement of the proof mass can be measured capacitively. In a capacitive sensing mechanism, the seismic mass is encapsulated between two electrodes. The differential capacitance is proportional to the deflection of the seismic mass between the two electrodes. The advantages of differential capacitive accelerometers are low power consumption, large output level, and fast response to motions. Better sensitivity is also achieved due to the low noise level of capacitive detection. Differential capacitive accelerometers also have DC response. Currently this kind of accelerometer has widely been used in most applications, especially in mobile and portable systems and consumer electronics.

### 2.2. Sensor Placement

Gemperle *et al.* [14] proposed the ergonomic guideline of “wearability” to describe the interaction between the human body and wearable objects. The “wearability map” was generalized to indicate the proper locations of a human body for unobtrusive sensor placement. These locations include the collar area, rear of upper arm, forearm, front and rear sides of ribcage, waist, thighs, shin, and top of the foot. These locations have common characteristics of similar area for men and women, a relatively larger continuous surface, and low movement and flexibility.

The sensor placement of wearable devices refers to the locations where the sensors are placed, and how the sensors are attached to those locations. Wearable activity sensors can be placed on different parts of a human body whose movements are being studied. In many cases, it is necessary to measure the whole-body movement. Therefore, the sensors are commonly placed on the sternum [15], lower back [3], and waist [16]. Most studies adopted waist-placement for motion sensors because of the fact that the waist is close to the center of mass of a whole human body, and the torso occupies the most mass of a human body. This implies that the accelerations measured by a single sensor at this location can better represent the major human motion. From an ergonomic point of view, the torso can better bear extra weight when carrying wearable devices. Sensors or devices can be easily attached to or detached from a belt around waist level. Therefore, waist-placement causes less constraint in body movement and discomfort can be minimized as well. A range of basic daily activities, including walking, postures and activity transitions can be classified according to the accelerations measured from a waist-worn accelerometer [16–18]. An approach using a chest-worn accelerometer was presented to detect respiratory and snoring features for apnea diagnosis during sleep [19].

Accelerometers can also be attached to wrists, thigh, or ankles. Sleep time duration can be determined from a wrist-worn accelerometer [20] and activity levels during sleep can be measured [21]. Ankle-attached accelerometers can significantly reflect gait-related features during locomotion or walking. Steps, travel distance, velocity, and energy expenditure can be estimated by an ankle-worn accelerometer [22,23]. A special placement in which an accelerometer unit integrated into hearing aid housing was used for detecting falls [24]. The rationale of this sensor placement was based on the author’s hypothesis that the individual intends to protect the head against higher acceleration caused by abnormal activities. Accelerometers have also been placed at the top of head for measuring balance during walking [25,26].

Another consideration for sensor placement is how to attach sensors to the human body. Wearable sensors can be directly attached to the skin [15,24], or with some form of indirect attachment by using straps, pant belts and wristbands, or other accessories [20,22,25,26]. Sensors and wearable devices can also be integrated into clothing [27]. In principal, the accelerometers or motion sensors should be securely fitted and attached to the human body in order to prevent relative motion between the sensors and the parts of the human body. Loose attachment or unsecured fit causes vibration and displacement of the wearable systems, and this is liable to produce extraneous signal artifacts and to degrade sensing accuracy.

## 3. Capabilities of Wearable Systems Using Accelerometry Measurement

Accelerometers can be used in ambulatory monitoring to continuously measure long-term activities of subjects in a free-living environment. The recorded longitudinal activity data can be used to identify postures and to classify several daily movements which are related to an individual’s functional status. Signal analysis and algorithm are used to classify daily human movements that are of interest, and adverse activity, such as falls can be detected as well. Important features extracted from posture sway and gait pattern have also been studied for the purposes of evaluating risks of falling and mobility. In addition, energy expenditure is the typical application featured by most commercially available accelerometers.

### 3.1. Posture and Movement Classification

Movement classification using accelerometry-based methodologies has been widely studied. Approaches to movement classification can be threshold-based or using statistical classification schemes. Threshold-based movement classification takes advantage of known knowledge and information about the movements to be classified. It uses a hierarchical algorithm structure (like decision tree) to discriminate between activity states. A set of empirically-derived thresholds for each classification subclass are required. Kiani *et al.* [28] presented a systematic approach to movement classification based on a hierarchical decision tree that enables automatic movement detection and classification. Mathie *et al.* [29] further presented a generic classification framework consisting of a hierarchical binary tree for classifying postural transitions, falling, walking, and other movements using signals from a wearable triaxial accelerometer. This modular framework also allows modifying individual classification algorithm for particular purposes.

Tilt sensing is a basic function provided by accelerometers which respond to gravity or constant acceleration. Therefore, human postures, such as upright and lying, can be distinguished according to the magnitude of acceleration signals along sensitive axes from only one accelerometer worn at the waist and torso [16,17]. However, the single-accelerometer approach has difficulty in distinguishing between standing and sitting as both are upright postures, although a simplified scheme with tilt threshold to distinguish standing and sitting has been proposed [16]. Standing and sitting postures can be distinguished by observing different orientations of body segments where multiple accelerometers are attached. For example, two accelerometers can be attached to the torso and thigh to distinguish standing and sitting postures from static activities [30–32]. Trunk tilt variation due to sit-stand postural transitions can be measured by integrating the signal from a gyroscope attached to the chest of the subject [33]. Sit-stand postural transitions can be identified according to the patterns of vertical acceleration from an accelerometer worn at the waist [17].

Acceleration signals can be used to determine walking in ambulatory movement. Walking can be identified by frequency-domain analysis [16,34]. It is characterized by a variance of over 0.02 g in vertical acceleration and frequency peak within 1–3 Hz in the signal spectrum [34]. Discrete wavelet transform is used to distinguish walking on a level ground and walking on a stairway [18].

Movement classification using statistical schemes utilize a supervised machine learning procedure, which associates an observation (or features) of movement to possible movement states in terms of the probability of the observation. Those schemes include, for example, *k*-nearest neighbor (kNN) classification [31,35], support vector machines (SVM) [36,37], Naive Bayes classifier [38,39], Gaussian mixture model (GMM) [40] and hidden Markov model (HMM) [41,42]. Naive Bayes classifier determines activities according to the probabilities of the signal pattern of the activities. In GMM approach, the likelihood function is not a typical Gaussian distribution. The weights and parameters describing probability of activities are obtained by the expectation-maximization algorithm. Transitions between activities can be described as a Markov chain that represents the likelihood (probability) of transitions between possible activities (states). The HMM is applied to determine unknown states at any time according to observable activity features (extracted from accelerometry data) corresponding to the states. After the HMM is trained by example data, it can be used to determine possible activity state transitions.

### 3.2. Estimation of Energy Expenditure

Energy expenditure (EE) can be estimated by measuring physical activities. The doubly labeled water method (DLW) and indirect calorimetry that measures oxygen uptake, carbon dioxide production and cardiopulmonary parameters are regarded as the gold-standard references of EE. Though accurate, gas analyzers for indirect calorimetry are expensive and they require specialized skills to operate. The isotopes analysis and production for DLW method are costly and are not suitable for large-scale studies [43]. Accelerometers provide an alternative method of estimating energy expenditure in a free-living environment. EE due to physical activity can be better predicted from the acceleration integral in anterior-posterior direction of an accelerometer [44], though vertical acceleration is most sensitive to major activities like walking or running. The signal integral of triaxial acceleration outputs has been found to have linear relationship with the metabolic energy expenditure due to several daily activities [45].

Commercial accelerometers usually convert the magnitude of accelerations to provide “activity counts” per defined period of time (epoch). The activity counts represent the estimated intensity of measured activities during each time period. Therefore, the recorded activity counts can be compared with questionnaires, or more accurately, the DLW method [46] or indirect calorimetry to estimate the energy expenditure due to activities [47]. Several regression equations can be derived or validated for different accelerometers to better match exact EE of physical activities among subjects.

Factors affecting the accuracy of EE estimation using accelerometry are the location and attachment of accelerometers, external vibration, gravitational artifact, and the types of activity performed in a free-living environment. Sensor attachment to trunk, lower back or second lumbar vertebra is preferred because the trunk represents the major part of body mass and moves with most activities. Gravitational effect is also relatively small on this body segment [45]. On the other hand, waist-mounted accelerometers are unable to measure upper limb movement and have inaccurate EE estimation when the subjects carry different loads of weight during activity [29]. Moreover, EE during walking may be inaccurately estimated when the locomotion is not horizontal, e.g., slope climbing and walking up and downstairs. A barometer that measures the atmosphere pressure was integrated with a triaxial accelerometer [34]. This approach can use the added information of altitude changes to determine movement with vertical displacement, such as taking elevator, walking upstairs and downstairs.

### 3.3. Fall Detection and Balance Control Evaluation

Fall-related injuries cause fracture and trauma which remarkably deteriorate the health and functional status of elderly people, leading to living dependence and higher risk of morbidity and mortality. Falls can be conceptually deemed as a rapid postural change from upright to reclining position to ground, or some lower level not as a consequence of sustaining a violent blow, loss of consciousness, sudden onset of paralysis as in stroke or an epileptic seizure [48].

The first approach to fall detection using accelerometry is published by Williams *et al*. [49], and a fall detector was presented after a number of pilot studies [50]. In its design implementation, the fall detector consisted of two piezoelectric shock sensors to detect the impact and a mercury tilt switch to identify the orientation. A two-stage detection process which detects both impact (acceleration) and orientation was used to better eliminate false alarms. The two-stage detection process firstly screens if any impact greater than a certain threshold exists (the first stage). A fall emergency is registered after the first stage if the reclining posture remains unchanged (the wearer does not get up) for a specific period of time. This design implementation led to the product commercialization of the fall detector by Tunstall Group [http://www.tunstall.co.uk/]. Similar approaches have been incorporated into fall detection algorithms using a waist-mounted accelerometer [16,17].

Lindemann *et al*. [24] evaluated a fall detector that was fixed behind the ear. Two high-g (50 g) accelerometers were orthogonally placed in the detector such that accelerations along all the sensitive axes could be measured. The fall detection algorithm used three trigger thresholds of sum-vector of acceleration in a plane (>2 g), the velocity before the initial impact (>0.7 m/s), and the sum-vector of acceleration in all spatial axes (>6 g) to recognize a fall. Though high sensitivity and specificity of the algorithm has been reported, such sensor placement would become an issue when ergonomics and integrated design of wearable system are considered.

Balance control or postural stability of the body while standing still or walking has been regarded as an important predictor of risk of falling of the elderly [51]. The physiological profile assessment (PPA) proposed by Lord *et al*. [52] also adopts postural sway as one of the six tests for screening risk of falling. In the balance test of PPA, postural sway can be measured using a sway meter that records body displacement at waist level. Force plate or pressure mat can be used to record the trajectory of center of pressure (COP) of body which also represents postural sway [53]. The postural sway measured from the sway meter and force plate shows strong correlation, and can provide similar information about balance sway.

Postural sway can also be measured by using accelerometers placed at the back of a subject [54–56]. Triaxial accelerometers have been used to obtain the postural sway projected on a level ground [57]. With the known height from the sensor to the ground, and the sensor output showing the tilt angle, trigonometric calculation can be applied to obtain the trajectory in anterior-posterior and medio-lateral directions projected on a level plane during a standing posture. The advantage of this technique is that the accelerometer is more sensitive to the difference of test conditions and is fully portable without the use of a force plate. Studies also showed a moderate correlation between trunk acceleration and COP pattern [58].

Significant gait parameters have been presented to assess balance control, functional ability, and risk of falling. Gait parameters during free walking can be measured by using accelerometers. Accelerometry data can be used to identify heel strike [59], gait cycle frequency, stride symmetry and regularity [60]. Measurement of temporal parameters of gait during long periods of walking using accelerometers was presented [61], and the spatial-temporal parameters were also measured using a miniature gyroscope [62]. Moe-Nilssen *et al*. [63,64] estimated the gait cycle characteristics of the subjects during timed walking. A triaxial accelerometer was attached to the lower trunk (the L3 region of the spine), and the signals were analyzed by an autocorrelation procedure to obtain cadence, step length, and gait regularity and symmetry.

Gait features between young and elder subjects have been compared by investigating accelerometry data. Vector magnitude (root mean square) values of accelerations obtained from the pelvis and head (vertical component) of elder subjects are smaller comparing with those obtained from young subjects [25,26]. Elder subjects showed slower velocity, shorter step length, and larger step timing variability during both walking on level and irregular surfaces from the temporal-spatial gait parameters between young and elder subjects. The harmonic ratio has been proposed as a measure of smoothness of walking, and is defined as the ratio of the summed amplitudes of the even-numbered harmonics to the summed amplitudes of odd-numbered harmonics both obtained from finite Fourier transform [65]. Older people with elevated risk of falling exhibited lower harmonic ratio [26].

## 4. Review of Current Products

There are many step counters available at very low prices that provide basic step counting and EE calculations. On the other hand, only a few commercial activity monitors use accelerometers. This section reviews several commercially available activity monitors using accelerometers, which are commonly used, compared and validated in research literatures, to provide a comprehensive outlook of current development status and how the activity monitors perform in various applications. Primary specification of the surveyed products are summarized and compared in Table 1.

### 

#### 

##### (1) SenseWear (BodyMedia Inc.)

The SenseWear Armband (BodyMedia Inc.,) is an activity monitor worn on the upper limbs to measure physical activities. The SenseWear Armband combines a dual-axial accelerometer to measure motion and multiple sensors to measure skin temperature, heat flux and galvanic skin response. This system can report the total EE, metabolic equivalent of tasks (METs), total number of steps, and sleep duration. The SenseWear armband was used in a weight intervention program [66]. Compared with other products and indirect calorimetry, the SenseWear armband accurately assessed EE across slow to normal walking, but showed underestimation of EE during increased walking speeds [67]. The SenseWear armband in connection with a fuzzy inference system was also used to distinguish motion states and emergency situations [68].

##### (2) CT1 and RT3 (StayHealthy Inc.)

StayHealthy Inc. has two motion monitor products, the CT1 Calorie Tracker and the RT3. Both products can be worn with a clip at the waist. CT1 is a FDA cleared Class II medical device for accurate EE estimation. RT3 is an activity monitor that uses a piezoelectric triaxial accelerometer to provide METs for clinical and research applications. RT3 also replaces the previous version Tritrac-R3D, which has been widely used in a number of studies and research applications.

A validation of RT3 for the assessment of PA reported that RT3 was a good measure of PA for boys and men [69]. RT3 has been used in recording temporal patterns of activity in chronic obstructive pulmonary disease (COPD) patients [70]. A study on the effect of a telehealth intervention for patients after coronary artery bypass surgery (CABS) used RT3 to measure PA and EE of the patients [71].

##### (3) AMP 331 (Dynastream Innovations Inc.)

The AMP 331 is an activity monitor positioned on the back of the ankle. With the proprietary “SpeedMax” technology, AMP 331 uses accelerometers to measure the forward and vertical accelerations to determine the position of the foot in space. Major gait parameters, such as stride length, speed and travelled distance during walking or running can be calculated. The recorded data in AMP 331 can be downloaded to PCs via a 916 MHz wireless radio receiver.

The company showed that the accuracy in distance computation is about 97% and even 99% after proper calibration. A study was conducted to validate the AMP 331 in assessing EE. This study recruited 41 subjects whose 12-hour daily activities in a field environment were recorded. The EE estimate from the AMP 331 and diary record were compared and the Pearson correlation coefficient is 0.651 [22]. The AMP 331 was reported to better estimate EE than other wearable sensors (comparing with the reference EE from indirect calorimetry) during walking with the manufacturer’s estimation equation [47]. The accuracy of the AMP 331 to detect atypical gait was also studied. The AMP 331 performed better than other sensors (comparing with data obtained from video recording) in detecting structured walking and stair ascent/descent [23].

##### (4) GT3X, GT1M (ActiGraph LLC)

The GT1M uses a uniaxial accelerometer and measures acceleration at 30 Hz sampling rate and 12-bit resolution in response to 0.05 to 2.5 g. The sampled signals are then bandpass-filtered between 0.25 to 2.5 Hz. The GT1M can be worn at the waist to measure activity counts, step counts, activity levels and EE. It can also be worn on the wrist for sleep monitoring. The data can be downloaded to the PC software “ActiLife” via USB connection.

GT1M has been used in evaluating PA levels in children and adolescents [72]. This device can accurately measure step counts and EE level between subjects in various ages [73]. de Vries [74] reported that the ActiGraph series was the most studied activity monitor, and many studies have validated its reliability and performance. The latest model GT3X uses a triaxial accelerometer for more accurate PA monitoring. GT3X is new and has been used in a study of physical activity in association with vascular function [75]. In addition, the company also releases ActiTrainer that uses the same triaxial accelerometer as that is used in GT3X, and a heart rate monitoring is integrated.

##### (5) StepWatch (Orthocare Innovations)

The StepWatch (also known as Step Activity Monitor, SAM) is an ankle-worn, microprocessor-controlled activity monitor for gait measurement. It records steps in a variety of gait styles and cadence. The StepWatch has also received FDA marketing clearance as a Class II device.

Foster *et al*. [76] investigated the accuracy in step counting of the StepWatch and found the negligible variance over all walking speed. It was reported to have minimum difference of step counts compared with the actual step counts during treadmill walking. The StepWatch showed better step counting at slow treadmill walking speed [77], but overestimated the steps during a 24 hour monitoring [78].

##### (6) activPAL (PAL Technologies Ltd.)

The activPAL is a motion sensor based on a uniaxial piezoresistive accelerometer. Worn and positioned on the thigh by direct adhesion to the skin, the activPAL classifies sitting, standing and walking among free-living activities. Recorded data is transferred to a PC via USB port. Ryan *et al*. [79] investigated the validity and reliability of the activPAL, showing that it was a valid and reliable tool in measuring step and cadence of the healthy subjects during walking. The activPAL was also compared with a discrete accelerometer device on the same healthy adults. The study indicated that the activPAL achieved a close match to the proven accelerometric data [80]. For older adults, the activPAL also exhibited accurate step counting and cadence compared with two other pedometers (New-Lifestyles Digi-Walker SW-200 and NL2000) [81].

##### (7) IDEEA (MiniSun)

The Intelligent Device for Energy Expenditure and Activity (IDEEA) is a device designed for PA and behavior monitoring, gait analysis, EE estimation and posture detection. An external set consisting of 5 biaxial accelerometers are attached to lower limbs and are wire-connected to a portable recorder worn at waist. It uses a 32-bit microprocessor that enables real-time data acquisition and processing. The IDEEA has been used in monitoring PA of obese people in real life environment [82], and has been validated in the study of ambulatory measurement for gait analysis [83], and EE estimation of PA [84].

## 5. Conclusions

Sensor-based measurement of human activities can provide quantitative assessment of physical activity. PA monitoring using accelerometry techniques enables automatic, continuous and long-term activity measurement of subjects in a free-living environment. All accelerometers provide basic step counting and activity counts (intensity) that can be used to estimate the energy expenditure due to PA. This has been widely adopted as an assistive method in the application of weight and dietary management. Postural sway can be measured by accelerometry that offer moderate correlation with reference to a force plate. Important gait parameters, such as the cadence, stride length, stride regularity, walking speed, can be measured using accelerometry to evaluate one’s risk of falling and mobility level. Detecting unusual movement, such as falling, is applicable to telecare or a personal emergency response system (PERS) for the elderly. In addition, accelerometry can assist traditional assessment tools for quantitative evaluation. For example, the TUG-T timing can be identified automatically according to the accelerometer outputs obtained from the test subjects. The time taken to perform each activity state can be objectively identified and the movement characteristics can be analyzed as well [85].

Approaches that utilize diverse sensors in a single accelerometer provide more activity information and may be expected to improve the accuracy in PA monitoring. Altimeters (pressure sensors) have been used along with an accelerometer to identify movements with altitude changes, such as walking up/downstairs. The ability to classify inclined walking may enhance the accuracy in EE estimation during PA. The measurements of human heat dissipation, skin temperature and conductivity have also been used in a commercial accelerometer-based activity monitor for accurate EE and metabolism rate assessments. In addition, accelerometers can be integrated into clothing from the ergonomics’ point of view.

In the future, the application of wearable accelerometry-based activity monitors should be provided with the integration to so-called “health smart home” monitoring systems [86]. Accelerometry data obtained from wearable accelerometers can be synchronized with the activity of daily living (ADL) data recorded by such monitoring systems to better describe the information of human mobility, physical activity, behavioral pattern, and functional ability that encompass the important parameters regarding the overall health status of an individual.

## Figures and Tables

**Table 1.** Product specification comparison.

	**SenseWear**	**CT1/RT3**	**AMP331**	**GT3X/GT1M**	**StepWatch**	**activPAL**	**IDEEA**
Size (mm)	88.4 × 56.4 × 24.1	71 × 56 × 28	71.3 × 24 × 37.5	38 × 37 × 18	75 × 50 × 20	53 × 35 × 7	70 × 54 × 17
Weight (g)	82.2	71.5	50	27	38	20	59
Accelerometer type	na	Piezoelectric	na	na	na	piezoresistive	piezoelectric
Number of accelerometer	1	1	2	1	1	1	5
Number of accelerometer axis	2	1/3	1 uni-axis and 1 dual-axis	3/1	2	1	2
Sensor placement	Upper arm	Waist	Ankle	Waist or wrist	Ankle	Thigh	Chest, thigh, feet
Sampling rate	32 Hz	0.017–1 Hz	na	30 Hz (12 bit)	128 Hz	10 Hz (8 bit)	32 Hz
Sensitivity range	2 g	na	na	0.05–2.5 g	na	2 g	5 g
Battery type	1.5 V AAA × 1	1.5V AAA × 1	na	3.7 V Lithium ion/Lithium Polymer	750 mAh Lithium	3 V li-polymer rechargeable	1 1.5 V AA
Battery life	3 days (continuous)	30 days	na	20 days	na	7–10 days	60 hrs
Data transmission	RF/USB	USB (docking tation)	916 MHz RF (USB wireless adapter)	USB	USB (docking station)	USB (ducking station)	USB
Data storage capacity	na	3 hours to 21 days (dependant on data resolution and collection)	na	16 MB (or 40 days)	2 months	na	7 days
Reported parameters	EE estimation, activity duration, sleep duration	Activity intensity, EE, MET	Steps, cadence, walking speed, stride length, distance, EE	Activity counts, steps, MET, activity intensity level	Steps gait characteristics	Sedentary and upright time, steps, stepping time, cadence, sit-to-stand activities, MET, PAL, kCal	Activity types, gait types, EE
